# Evaluating the Implementation of a Mental Health Referral Service “Connect to Wellbeing”: A Quality Improvement Approach

**DOI:** 10.3389/fpubh.2020.585933

**Published:** 2020-12-14

**Authors:** Leigh-ann Onnis, Irina Kinchin, Josephine Pryce, Priscilla Ennals, Joe Petrucci, Komla Tsey

**Affiliations:** ^1^The Cairns Institute, James Cook University, Cairns, QLD, Australia; ^2^College of Business, Law and Governance, James Cook University, Cairns, QLD, Australia; ^3^Centre for Health Economics Research and Evaluation, Centre for Improving Palliative, Aged and Chronic Care through Clinical Research and Translation, University of Technology Sydney, Sydney, NSW, Australia; ^4^Trinity College Dublin, The University of Dublin, Dublin, Ireland; ^5^Neami National, Preston, VIC, Australia; ^6^Neami National, Cairns, QLD, Australia; ^7^College of Arts, Society & Education, James Cook University, Cairns, QLD, Australia

**Keywords:** mental health service, process evaluation, referral service, outcomes evaluation, quality improvement, participatory action research (PAR)

## Abstract

There is increasing demand for mental health services to be accessible to diverse populations in flexible, yet, cost-effective ways. This article presents the findings from a study that evaluated the process of implementing Connect to Wellbeing (CTW), a new mental health intake, assessment and referral service in regional Australia, to determine how well it improved access to services, and to identify potential measures that could be used to evaluate value for money. The study used a hybrid study design to conduct a process evaluation to better understand: the process of implementing CTW; and the barriers and factors enabling implementation of CTW. In addition, to better understand how to measure the cost-effectiveness of such services, the hybrid study design included an assessment of potential outcome measures suitable for ascertaining both the effectiveness of CTW in client health outcomes, and conducting a value for money analysis. The process evaluation found evidence that by improving processes, and removing waitlists CTW had created an opportunity to broadened the scope and type of psychological services offered which improved accessibility. The assessment of potential outcome measures provided insight into suitable measures for future evaluation into service effectiveness, client health outcomes and value for money.

## Introduction

There is increasing demand for health service organizations to provide accessible mental health services to diverse populations across vast geographical areas in flexible, yet, cost-effective ways. With the Global Burden of Disease study reporting that depression and anxiety have high societal costs and contribute toward increased levels of disability, new approaches to providing cost-effective treatment for these high prevalence disorders are needed (1, 2). There is growing momentum toward therapies offered within a stepped care framework where clients are directed to services that meet their needs (3, 4). In essence, stepped care is a model which seeks to improve access to health services through better allocation of scarce resources (5). Stepped care services have the potential to reduce wait times for clients; avoid unnecessary use of high-needs/high cost mental health services; and decrease the burden on the health system overall. Richards [(4), p. 210] suggests that to be successful stepped care services need: a high level of clinical outcome data, suitably trained staff, equity of access, and a focus on reducing attrition at each stage of the system (e.g., referral-to-assessment; assessment-to-treatment; treatment-to-discharge). Our literature review uncovered an absence of studies that contained empirical evidence about how to collect the necessary high level outcome data, improve equity of access and reduce attrition in “real world” mental health services. This is consistent with the findings from other studies which report a scarcity of empirical evidence about “implementation, training, policy, or key success factors” for stepped care mental health services [(6), p. 8]. Therefore, there is a need for more empirical evidence to improve our understanding of suitable clinical outcomes data available in routinely collected clinical data, improving equity of access, and how to reduce attrition as clients progress through the system.

Further, Richards (4) reported that while the evidence about the effectiveness of stepped care is largely accepted; little is known about the best ways of organizing the provision of psychological therapies so they are equitably available and widely used by clients. Similarly, Dalton et al. (6) found that information (e.g., costs, referral systems) about effectively operating low intensity interventions within a stepped care model was missing from the literature. Furthermore, Richards et al. [(5), p. 9] argued that “Access to care has not traditionally received the same research focus as issues of treatment effectiveness.” Moreover, in countries, such as Canada and Australia, where populations live over vast geographical areas, healthcare resources tend to be concentrated in urban areas, particularly for expensive psychological therapies. Therefore, more research is required to understand equity in access challenges with economically, physically, and socially disadvantaged groups experiencing poorer access to health care than those living in urban areas (6).

One organization that is developing innovative ways to meet the needs of clients with low incomes living in regional areas (urban, rural, and remote) is Neami National. Neami National's Connect to Wellbeing (CTW) is providing an intake, assessment, triage and referral service which aims to connect people with the right service at the right time. This approach aims to simplify access to psychological services for people, who may otherwise have difficulties in accessing mental health services. Based on the stepped care model of mental health support, CTW focuses on integration of the health care system and facilitates the matching of people with services to meet the need identified at the time of referral. The only description in the literature of a service that may be comparable to the approach used by CTW, was one of the sites in the evaluation of four early implementer sites in the UK (5). In the early implementer site study, the authors described each site saying that, at three sites, therapeutic services could be provided by the organization; however, at one site, several different organizations could provide different elements of the psychological therapies service. Although it is not described in any further detail, the last site appears to have a similar operational model to CTW, in that they receive referrals, assess and then connect clients to suitable services offered by another service provider (5). Unfortunately the study only reports the aggregated data (collated for all four sites), and therefore cannot inform this evaluation of the implementation of CTW. A scoping literature review was unable to identify any other comparable services revealing a shortage of academic literature on implementation and empirical evaluation of these types of “real world” health referral services.

Services, such as CTW need to be cost-effective as well as accessible. However, it is difficult to conduct cost-effectiveness studies without dedicated funding (7, 8). Hence, it is advantageous if routinely collected data generated through the care of clients, could also be used for outcome evaluation data. Our review found that the measures most commonly used for measuring client outcomes and cost-effectiveness were the EuroQoL Five Dimension Five Level Version (EQ-5D-5L), the Recovering Quality of Life 10 item scale (ReQoL-10) and the Client Service Receipt Inventory (CSRI) (1). Some studies used pre-post questionnaires for their economic argument which was based on the assumption that clients show sustained recovery which results in economic benefits (9). Economists and clinical researchers both argued that increased access to psychological therapies would “largely pay for itself” by reducing related public costs (e.g., welfare benefits, health system costs) and through increasing revenues (e.g., employment related taxes, increased productivity) (2, 10).

In summary, the review of the current literature revealed that accessibility to service, clinical outcomes data, and reducing attrition within the system, as well as the cost-effectiveness of the service are all important. Unfortunately, the review did not identify any evaluations of mental health services referral hubs similar to CTW, which highlights the contribution a study such as this one to the scarcity of empirical evidence (6).

### Research Questions

This study sought to evaluate CTW in real time, as it was being developed. As such, the systems and processes were still being developed which created some limitations for the type and amount of client data that could be collected. Therefore, our research questions focused on the process of implementing CTW, and what we could learn during the implementation process for future research about patient outcomes and cost-effectiveness.

1. What are the barriers to, and factors enabling the process of implementing the CTW service?
2. What outcome measures can the CTW service use in routine data collection to inform a future service effectiveness study?
3. How can insights gained from the CTW experience inform future service development?

## Methods

The study methodology was informed by the recent development of the evaluation framework for a Queensland Mental Health Prevention and Recovery Care service using quality improvement (QI) approaches (11). QI is characterized by the iterative use of processes and is increasingly being used in the evaluation of health services to bridge gaps between the evidence base for best practice, what actually happens in practice, and the achievement of improved population health outcomes (12). Participatory Action Research (PAR), which is based on reflection, data collection and taking action for change and improvement is the QI approach used in this evaluation (13).

The evaluation was conducted over an 18 month period (July 2018 to December 2019). The aim was to conduct a process evaluation using the CTW experience to better understand: (a) the process of implementing the new service; and (b) the barriers and factors enabling implementation of the new service. While the process evaluation was the focus of this study; a complementary investigation was conducted to identify potential outcome measures that CTW can collect through routine data collection processes to undertake economic evaluation or value for money analysis of the CTW service in the future.

### Study Setting

**Figure 1:**
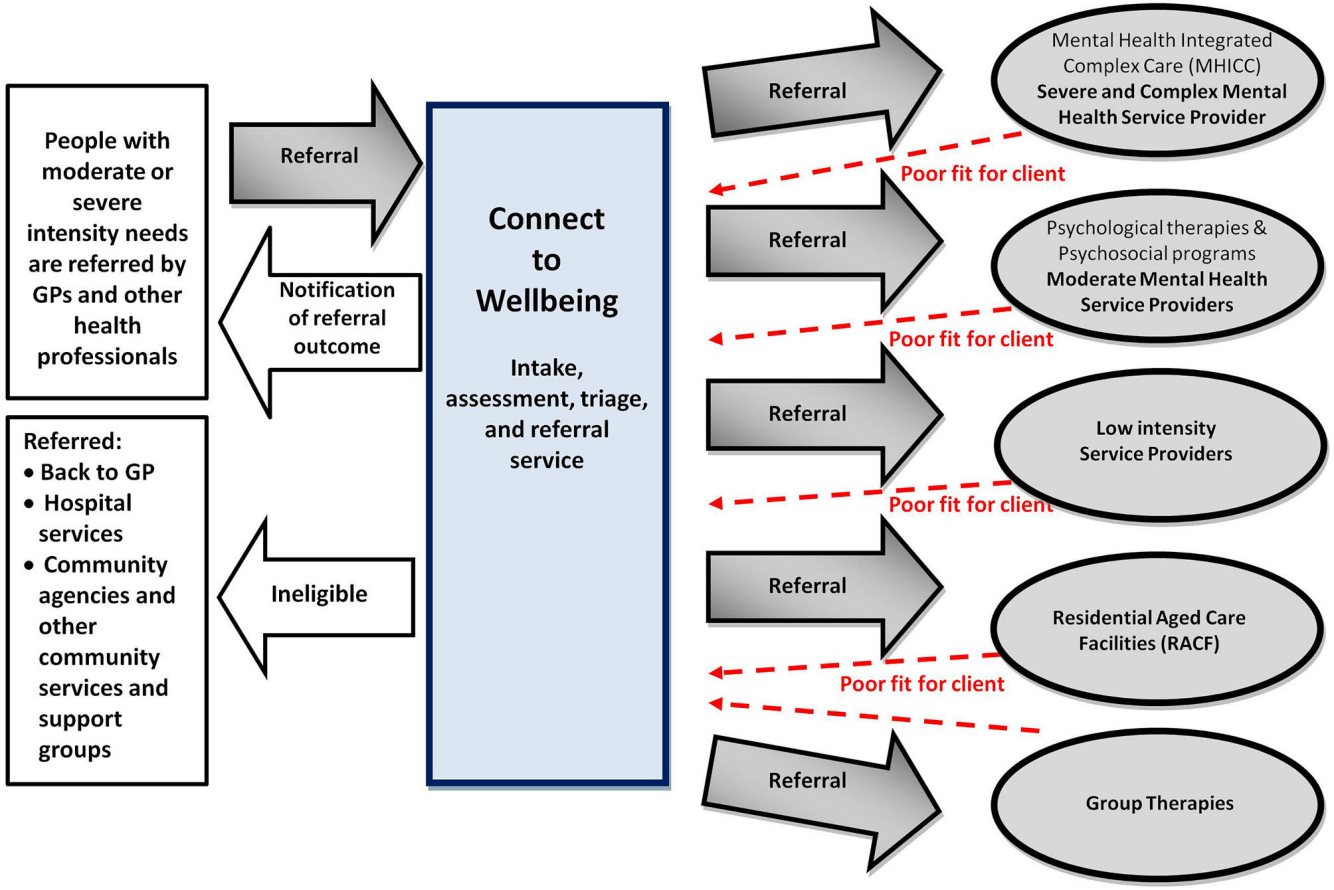
Connect to Wellbeing's role in referral pathways for stepped care mental health services.

CTW is an Initial Assessment and Referral (IAR) service, developed to improve access to psychological services to people on low incomes, and to address the need for effective systems for referral. CTW refers people within a stepped care model, whereby, a person is matched to the least intensive level of treatment needed. The role that CTW plays in the referral pathway is depicted in Figure 1. Prior to the establishment of CTW, General Practitioner (GP) referrals were usually sent to one of the three providers, each of whom would complete an intake process and where possible arrange for the individual to access psychological therapy; however, geographical and funding limitations often resulted in clients being waitlisted until services were available. CTW offers an equitable system of accessing services because CTW sits between the GP and the service provider, and therefore, can match the client needs and the capability of the service providers in real time (i.e., which services have the capacity for the client at the time). This is a more objective, transparent allocation process than the previous arrangement where the GPs referred directly to service providers, a model which resulted in waitlists. People are eligible to receive up to six sessions of psychological therapy, with potential for an additional six sessions, dependent on need.

**Figure 2:**
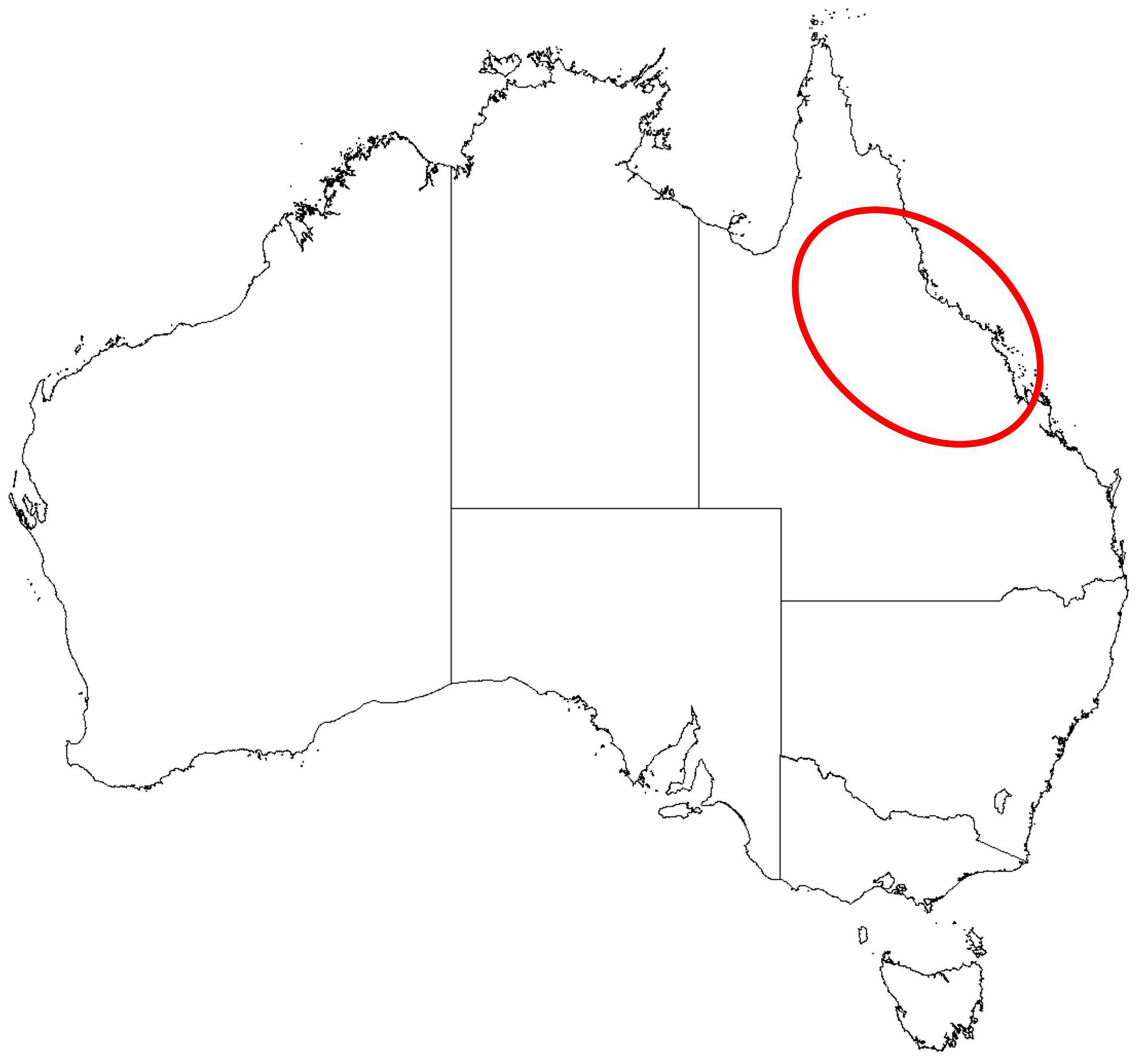
Map of the area covered by Connect and Wellbeing.

The North Queensland Primary Health Network (NQPHN) administers the funding for mental health services, including the CTW referral service across north eastern Australia. The region is a large area of ~381,264 square kilometers (14–16). The area serviced by CTW extends from just south of Mackay all the way up the coast to Cow Bay in the north, and extends inland to just west of the rural towns of Croydon and Richmond (Figure 2). The area includes coastal populations, farming and agricultural industries, islands and isolated communities, and regional centers. Across this large diverse geographical area, CTW provide services to an estimated resident population of 662,173 people (14–17). Based on 2016 census data, the majority (60.96%) of the population is aged 19–64 years; and approximately one quarter (25.87%) are 18 years or younger (18). In 2016, it was estimated that 10.1% of residents within the NQPHN region (including Cape York and the Torres Strait Islands) were Aboriginal and/or Torres Strait Islanders (19).

The NQPHN region contains the most decentralized population in Australia, with “subpopulations characterized by elevated risk of negative mental health outcomes” [20, p. 11]. In the NQPHN region, there are general challenges to accessing services that many populations experience with similar geography (e.g., fewer services in rural and remote areas); however, there are also less common additional barriers to accessing psychological services (e.g., high rates of homelessness, lack of bulk billing through Medicare for General Practitioners (GPs) in some rural areas) (20).

### Research Design

This study used a pragmatic uncontrolled mixed methods hybrid design that sought to jointly describe implementation from multiple perspectives. While the focus was investigating the implementation process through the hybrid design, data were also analyzed to identify potential outcomes measures. The advantage of hybrid research design is that by simultaneously assessing the implementation process and conducting an assessment of potential outcomes measures, the hybrid design can speed up and improve research translation, and increase the usefulness and policy relevance for decision-makers (21).

The key elements of the hybrid design were a time series analysis combined with thematic analysis to understand the process of service implementation; and an investigation into potential outcome measures using a before and after assessment approach testing the feasibility of relevant self-reported outcome measures. In addition, the collaboration explored the possibility of conducting a cost assessment of running the CTW service (pending data availability). The cost assessment was not conducted.

The evaluation process included: planning the evaluation, literature reviews, and the formation of an Evaluation Steering Group. Consistent with Participatory Action Research an Evaluation Steering Group involving people directly impacted by the service implementation, was formed to oversee the evaluation. This group included four people with lived experience of mental illness as consumers or carers, CTW staff and managers, a NQPHN representative, and university-based researchers. See Supplementary Table 1 for the steps in the evaluation process and Supplementary Figure 1 for the Evaluation plan.

### Data Collection and Analysis

#### Process Evaluation

The data for the process evaluation included: transcripts from four Evaluation Steering Group meetings (July 2018, December 2018, May 2019, and August 2019), three quarterly reports produced by CTW, and the Your Experience of Service Survey (YESS). CTW sent a YESS to nearly 2,000 people in March 2019. The YESS contained an opportunity for client feedback using the statement, “If you would like to include any comments, please write them here.” Of those returned, 125 (6% response rate) included written responses. While the response rate was low, it was not being used to assess the effectiveness of CTW, rather to show improvements and was, therefore, considered sufficient to test proof of concept. The small sample size is acknowledged as a limitation.

The qualitative data were analyzed using NVivo 12. First a thematic analysis of the YESS responses was conducted with free coding used to identify emergent themes by co-author LO. The emergent themes from the YESS survey were presented and discussed at the Evaluation Steering Group Meeting in May 2019. Then, a thematic analysis of the transcripts and reports was conducted using Nvivo 12 by co-author LO, and were presented and discussed at the Evaluation Steering Group Meeting in August 2019. The data for the time series analysis were analyzed by CTW and reported in the Quarterly Service Reports. The reduction in the waiting list and the increasing referrals captured in the time series analysis were also an emergent theme in the thematic analysis. The findings from each thematic analysis and the time series analysis were presented and discussed at the final Evaluation Steering Group Meeting in April 2020. The Evaluation Steering Committee Members and all co-authors of this paper agreed on the final themes.

#### Identifying Potential Outcome Measures

While not the main focus of the study, under the hybrid study design routinely collected data were assessed as potential outcome measures. As CTW were developing the service during the process evaluation, there was an opportunity for researchers to discuss and inform CTW about survey tools that were both clinical in nature and provided suitable data for an outcomes evaluation. Several options were considered before settling on two health related quality of life measures (EQ-5D-5L and ReQoL-10). The Evaluation Steering Group also identified employment and absenteeism as potential areas of impact of CTW. Therefore, researchers added three additional questions about employment.

The EuroQol Five Dimension (EQ-5D) was used because it is the shortest (five questions) and the most widely used generic measurement of quality of life across five domains (mobility, self-care, usual activities, pain/discomfort, anxiety/depression) (22). Updated to include five levels of severity for each domain, the EQ-5D-5L is extensively used in the clinical and economic evaluation of health care and is helpful for comparing results across different studies and settings (22–24). Most importantly, EQ-5D-5L is not disease-specific, and therefore, applicable to most disease areas and comparisons of interventions across disease areas. Further, the EQ-5D-5L assesses mental as well as physical health states which is a strength given known links between mental and physical health.

Despite limitations, there were sufficient data to assess the EQ-5D-5L, ReQoL-10 and a customized set of questions as potential outcomes measures. The analysis commenced with the respondent's EQ-5D-5L scoring which was analyzed descriptively, as well as converted to a single summary index score reflecting societal preference compared to other health profiles. Societal preference weights were derived using a valuation set from the Australian general population (25). Index scores range from <0 (where 0 is the value of a health state equivalent to dead; negative values representing values as worse than dead) to 1 (the value of full health), with higher scores indicating a better health profile.

Recovering quality of life (ReQoL-10) is a new Patient Reported Outcome Measure that has been developed to assess the quality of life for people with different mental health conditions (26). The ReQoL is a condition specific measure, and while it somewhat resembles a well-being measure, it was developed specifically to measure mental health recovery. The ReQol-10 contains 10 mental health questions and one question about physical health. An overall ReQoL-10 index score was calculated by summing the numbers for the 10 questions. The minimum score is 0 and the maximum is 40, where 0 indicates poorest quality of life and 40 indicates the highest quality of life.

Employment and absenteeism were measured using three questions:

1. What is your current occupational status?
2. How many DAYS have you been absent from work/usual activities (paid or unpaid) due to illness in the past 4 weeks (28 days)?
3. How many HOURS are you expected to work/perform usual activities in a typical 7-days week? If it varies, estimate the average.

The data is presented in the results section to show changes in health or employment before and after. The significance level was set to 0.05 or 5%.

A CTW staff member contacted new referrals and collected the outcomes data over the telephone for the first survey. For the second survey, the list of participants from the first survey was monitored and participants were contacted by a CTW staff member once they had received the psychological therapy for which CTW had referred them (between 3 and 6 months after the first survey).

### Ethics

Ethics approval for this evaluation was received from the James Cook University (JCU) Human Research Ethics Committee (HREC) (#H7549).

## Results

### Process Evaluation

The thematic and time series analysis revealed seven emergent themes:

1. Developing and improving systems,
2. Referrals and Waitlists (access to services),
3. Workforce and workload challenges,
4. Responding to the impact of external changes,
5. Type of psychological services offered,
6. Demand management, and
7. Referring to the right service while there is increasing demand for services.

The QI approach comprised an iterative process of reviewing and implementing improvements in real time, and as such did not follow a straight line from creation to improved accessibility. Similarly, the findings from the process evaluation cannot be described in a straight line from the first day to the end of the 18 months period of this study. Therefore, this section uses these emergent themes as a narrative to describe the key aspects of the process of implementing the new CTW service. The section concludes by using the CTW experience to highlight the barriers and enablers from the empirical study as a means to inform others considering a similar mental health referral service model.

#### Developing and Improving Systems

The establishment of CTW offered a system that streamlined the process for accessing psychological services Yet, despite stakeholder and community consultation reinforcing the need for a referral service, such as CTW, when CTW commenced they were not sure how well the new service would be received, as one CTW staff member explained, “When we first started there was a fear that we were an extra service in the middle, between what had previously been straight forward … we might be seen to be interfering in what used to be easier.” However, the referrals continued to come in, and through education about the referral process the transition from three different providers to CTW was successful and since their launch in June 2018, referrals have continued. While it is not possible to determine whether the increase is driven by the improvements arising from the establishment of CTW or by greater community needs; the continued usage of the referral process suggests that the transition went well.

When establishing the three sites, it was necessary to develop systems to adequately support the needs of CTW management, staff, and stakeholders across all the sites. As CTW started from scratch, they created and updated their policies, procedures and systems as CTW evolved. Unfortunately, this created additional challenges for CTW staff who found it difficult to keep up with the changing processes with one CTW staff member saying that, CTW should have “Take[n] more time to implement processes prior to commencement.” While there was some frustration from staff with the implementation process, it would appear reasonable given the challenges of implementing a new service.

Although CTW started from scratch, they inherited some existing databases and data management systems. During the 18 months of operation CTW had to manage multiple changes to the data management system. There were issues with the incompatibility between systems and some aspects of the client data management system were inadequate for their reporting requirements which resulted in some manual data analysis. These issues were not completely resolved during the establishment of CTW; however, they are improving and CTW management report that they are more reliable than previously.

#### Referrals and Waitlists (Access to Services)

**Figure 3:**
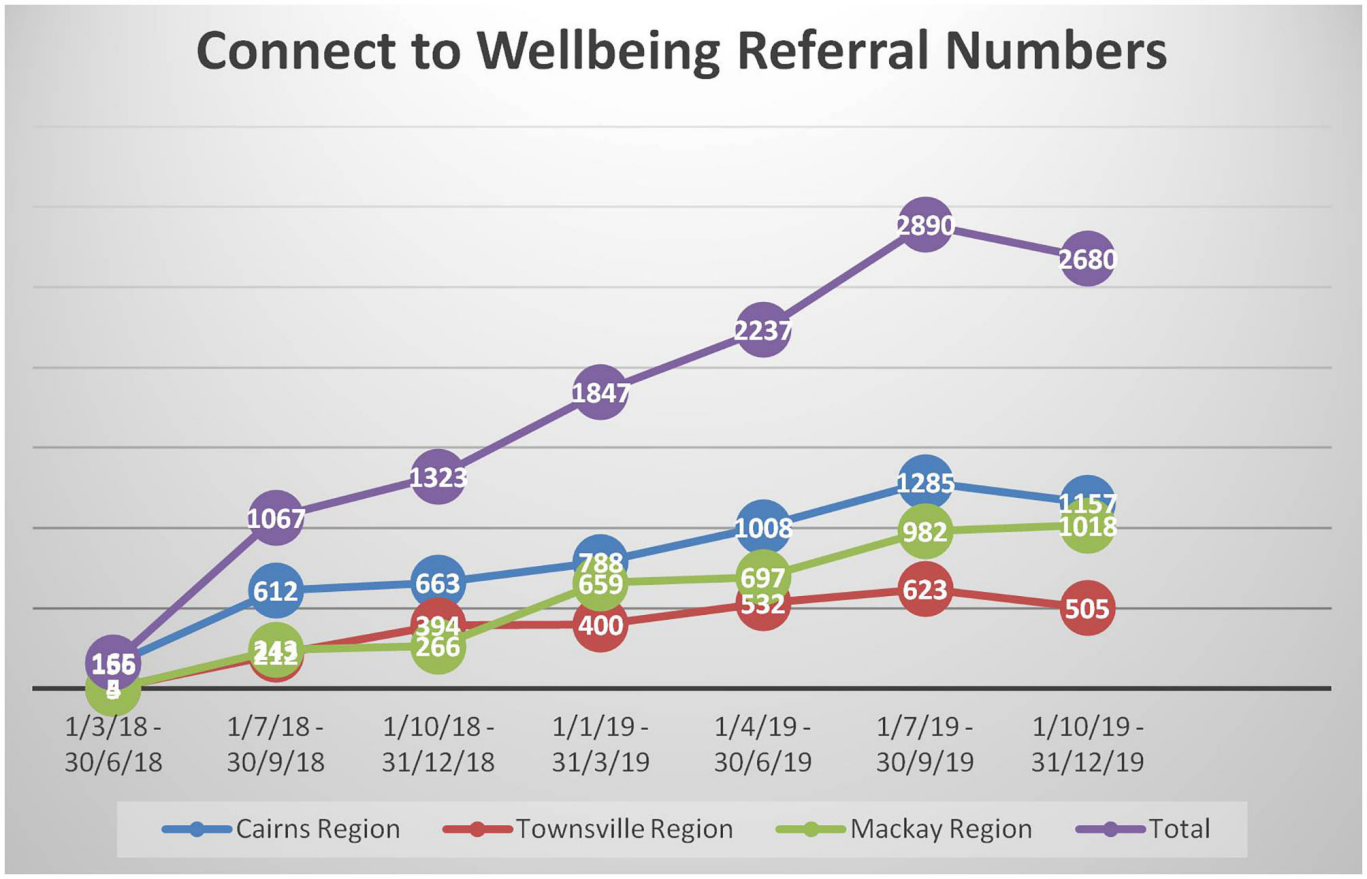
The number of referrals for each of the connect to well-being sites (period ending 30 June 2018 to period ending 31 December 2019).

When CTW commenced there were long waitlists to access services; 558 people, many of whom had waited up to 1 year at two sites, and at the third site people had been declined access to services. Clients commented on the long wait times (e.g., “the long processing time from when my doctor sent the referral to when Connect to Well-being contacted me …”). The waitlists have gone, no-one who is eligible is being declined access to services, and in December 2019, 18 months since the CTW service began, the waitlists had not returned. Since clearing the waitlists, CTW has successfully managed demand in an environment where each quarter has seen significant increases in referrals (Figure 3). When CTW was first implemented (April–June 2018) there were only 155 referrals, for the next period (July–Sept 2018) this increased significantly to 1067 referrals and it has been increasing ever since [CTW Quarterly report (April–June 2019)]. In the period July–September 2019, there were 2890 referrals followed by 2680 referrals in the final quarter of 2019 [CTW Quarterly report (July–September 2019); (October–December 2019)]. While there appears to be fewer referrals in the October–December 2019 period, this includes the Christmas period, a time where service provision traditionally slows down as many service providers have leave [CTW Quarterly report (October–December 2019)]. This is consistent with the data in the October–December 2018 period where the number of referrals was fewer than the preceding and following periods.

The data shows that more than 6,000 referrals were managed in the financial year, 1 July 2018 to 30 June 2019 [CTW Quarterly report (April–June 2019)]. The absence of waitlists not only means more timely referrals for clients; it means that clients for whom low intensity services are not suitable can be referred to psychological services more suited to their needs. As one CTW clinician explained:

*There were so many people on the waitlist who were not necessarily appropriate for service … So you had all these people that were waiting and if they had been spoken to initially, in what we [CTW] do through assessment I guess they could have referred to the right program and could have received treatment straight away. So that has been a huge bonus and a massive improvement*.

CTW has undertaken service mapping across the region to better understand the distribution of service providers and areas where there are barriers to accessing services. In some areas, there is an absence of providers in some types of services (e.g., children under 12) and/or an absence of bulk-billing GPs which reduces the number of referrals to CTW. Despite the initial challenges CTW has managed to improve access through innovative solutions, such as using specialist Telemedicine services, or engaging with other non-profit organizations in regions where there is limited access to providers of the type of psychological service needed.

#### Workloads and Workforce Challenges

The continued increase in demand has impacted the CTW workforce. Workloads have increased significantly and are increasingly challenging to manage. In response, CTW management reported that “Casual staff are used on occasion to attend to demand issues, and increasingly the use of the casual staff is becoming integral to the ongoing functioning of the teams” [CTW Quarterly report (July–September 2019)]. While workforce flexibility reduces the pressure to some extent, the significant increase in demand for services had continued to put pressure on CTW's available resources. As a result, CTW staff have innovative solutions to manage the high workloads and to meet their site-specific issues which, while largely effective, have created inconsistencies in practice across sites. Governance, monitoring systems, and improved communication are being used to ensure uniformity across the three sites. However, ensuring consistency across the three sites remains a challenge for CTW management.

#### Responding to the Impact of External Changes

Since commencing, CTW has been impacted by several significant events. An extreme flooding event “had a major/devastating impact on the city of Townsville and the rural communities west of Townsville” and directly impacted staff (CTW Quarterly report, April–June 2019, p. 3). Then, the sudden closure of a large provider of psychological services saw CTW commence contracting with service providers directly. In one of the three regions this required a change from CTW's original operating model as an intake and referral service, and is one way in which CTW has been able to respond to a significant external impact, adapting to continue to provide clients with access to suitable services. In addition, CTW has integrated the Mental Health Integrated Complex Care (MHICC) program into the Stepped Care Continuum and has been working to adapt to external changes and continue to improve services (e.g., referrals to psychological therapies in Residential Aged Care Facilities commenced in 2019).

#### Type of Psychological Services Offered

Not only has CTW been adapting and responding to external changes, they have been seeking innovative and cost-effective ways to provide services to all clients. Where there are fewer services available CTW were able to utilize online services available which supported referrals to suitable specialist Telehealth services. In addition, CTW have supported service providers to establish group therapy. Despite the challenges and potential barriers in implementing group therapy (e.g., venue, financial viability), CTW have made efforts to support service providers, as one CTW clinician explained:

*Group therapies have started and for me that is pretty amazing because there haven't been group therapies in the program in North Queensland. There are a couple of groups off the ground and its running, and I feel that is going to gain some momentum. And in years to come that will give us a good basis and really change the trajectory of the service delivery because I think that it is better if we do a lot more group work rather than individual work*.

By late 2019, CTW was established in North Queensland, and were working with health service networks, community groups and other government services. Also, CTW anticipate that improved engagement with Aboriginal and Torres Strait Islander organizations will improve access to suitable services for Indigenous clients. In addition, CTW have identified opportunities to better engage with organizations, such as Headspace, EdLink, and other private providers to better meet the needs of clients across all age groups, particularly young people under 12 and older people in aged care.

#### Demand Management

While CTW is continuously considering its demand management processes and adapting to meet client needs, the extent to which this demand will shape CTW is unknown. According to the CTW Quarterly report (July–September 2019, p. 2), “the ultimate aim is ensuring people can access services when they need to, and ensuring they are receiving the right service relative to their needs” however they report that, “the challenge of the demand management is driving necessary reform to the Stepped Care program.” By the end of 2019, the continued increase in referrals, the increased workload and subsequent workforce challenges, and the lack of providers for some specialist services in particular locations was putting pressure on CTW managers and staff. CTW has continued to adapt processes and systems; however, they recognize that more long-term solutions are required with the continued increase in demand for services.

#### Referring to the Right Service While There Is Increasing Demand for Services

CTW appears to be nearing the end of the establishment phase of the service. The continued demand for services and the continued absence of a waitlist suggests that 18 months into operation, the implementation of CTW has been successful. Unfortunately, CTW were unable to collect feedback from GPs during the study period; however, some indirect feedback was received and discussed at Steering Group Meetings. An excerpt from an email received by the CTW Regional Manager stated that, “My first client was very appreciative of our service. She stated that her GP [name redacted] highly recommended our service and stated that we always ensure we refer our clients to the most suited provider.” In the absence of direct survey feedback from the GPs, for this evaluation, the positive GP sentiment described by clients, the disappearance of the waitlists, together with the continued increase in referrals and the absence of complaints from GPs is taken as a positive response to the CTW service.

The YESS feedback also suggests that CTW are achieving their aim with one client saying “I am grateful for the referral to this service and believe it has the potential to help with my conditions,” and “Since moving over to a different provider I feel more suited to, I now feel I get a lot of support.” These comments suggest that clients are satisfied with the CTW service. One even provides an example where the first service provider offered was not right, so CTW helped to find the right service for them. This sense of satisfaction was also described by a CTW clinician,

*I think a real highlight is when you say to the clients, when you have a conversation about what is the best service for them and they thank you and they are just so happy and really feel like you can do that service for them*.

Overall, CTW have developed the systems, policies and processes needed to achieve its objective of ensuring the right people, receive the right service, at the right time. This was supported by a CTW manager who said that one of the highlights of their day was when they hear clinicians discussing complex referrals in an effort to find the right service for the client, saying:

*Getting that person on the right path, there wasn't a mechanism for that, to really try to figure out really what this person needs and then to hear about all the machinations in their thinking and thoughts and the lengths they go to, really, is really positive in my mind that they are really trying to get this person to the right service. Which I think is just fantastic. I love the effort that they give it*.

### Barriers and Enablers

The thematic analysis revealed the emergent themes, which in turn highlight the key aspects of the CTW service implementation. In addition, they provide insight into the barriers and enablers for implementing a service, such as CTW.

#### Barriers

- Capacity of service providers to meet demand for services
- Incompatible data management systems
- Not having procedures, processes and systems in place early enough
- The lack of providers in some types of services; and in some geographical areas
- Environmental impacts (e.g., flood event, closure of service providers).

Enablers

- Knowing and having capacity to influence data management system from the outset
- Rapid feedback processes from stakeholders (e.g., the Evaluation Steering Group, YESS)
- Ability to adapt to internal and external impacts
- Workforce flexibility (e.g., casual workers)
- Educating/training GP's and/or referrers in the referral process
- Building relationships with other service providers
- Innovative solutions to managing demands (e.g., Telehealth, site specific solutions, Group Work).

### Assessment of the Potential Outcome Measures

In addition to the process evaluation, this study included an assessment for potential outcome measures. This section contains an overview of the analysis on the data collected for the potential outcome measures. These findings are being reported in terms of their value as potential indicators, not as measures of the effectiveness of CTW during the period in which they were collected.

A baseline assessment was completed with data from140 CTW clients, 65 of whom completed the follow-up assessment. At the follow up, the majority of clients were females (65.2%) with an average age of 43.7 years.

The average number of interactions with the therapeutic service providers they were referred to by CTW was 3.7 sessions (ranging from 0 to 17). Eleven people out of 65 (17%) did not receive support from the first provider they were referred to through CTW. People might not receive the support they required for several reasons, including being on a waitlist (inherited when CTW commenced), a lack of relevant services being available in their area, or the person may have chosen not to continue. In these cases, it was considered a negative outcome if the person was deemed by their GP to need psychological services but did not receive a service. In contrast, a referral was made to another service provider for 11 CTW clients (17%), including eight people who received referral to an alternative provider of psychological therapies and three who were referred for counseling (outside the CTW service provider network). The perceived enhanced “fit” of services to client need was a feature of the CTW stepped care model and, therefore, being referred outside the CTW network was viewed as a positive outcome for CTW.

#### Generic Quality of Life (EQ-5D-5L)

Between baseline and follow-up, EQ-5D-5L showed “mixed” changes in health: better on one dimension, but worse on another. For example, fewer people felt extremely or severely anxious or depressed and felt less extreme pain or discomfort compared to the baseline assessment. However, more people indicated problems with mobility, self-care or usual activities at the follow-up assessment. Table 1 shows significant differences in the distribution of responses to the mobility, self-care, anxiety and depression dimensions of the EQ-5D-5L (*p* < 0.05, < 0.05, and < 0.001, respectively).

**Table 1:** Distribution of EQ-5D-5L dimension responses at baseline and at follow-up.

**Dimension**	**Baseline *n* = 140 (100%)**	**Follow-up *n* = 65 (100%)**	*p*-value^*^
**Mobility**			
No problems	98 (70.0%)	39 (60.0%)	*p* <0.05
Slight problems	21 (15.0%)	8 (12.3%)	
Moderate problems	11 (7.9%)	15 (23.1%)	
Severe problems	3 (2.1%)	3 (4.6%)	
Unable to walk about	2 (1.4%)	0 (0.0%)	
**Self-care**			
No problems	126 (90.0%)	48 (73.8%)	*p* < 0.05
Slight problems	5 (3.6%)	9 (13.8%)	
Moderate problems	4 (2.9%)	7 (10.8%)	
Severe problems	0 (0.0%)	1 (1.5%)	
Unable to wash or dress	0 (0.0%)	0 (0.0%)	
**Usual activities**			
No problems	79 (56.4%)	34 (52.3%)	*p* = 0.144
Slight problems	27 (19.3%)	8 (12.3%)	
Moderate problems	23 (16.4%)	21 (32.3%)	
Severe problems	5 (3.6%)	1 (1.5%)	
Unable to do usual activities	0 (0.0%)	1 (1.5%)	
**Pain/discomfort**			
No pain/discomfort	44 (31.4%)	26 (40.0%)	*p* = 0.648
Slight pain/discomfort	44 (31.4%)	10 (15.4%)	
Moderate pain/discomfort	33 (23.6%)	17 (26.2%)	
Severe pain/discomfort	9 (6.4%)	10 (15.4%)	
Extreme pain/discomfort	5 (3.6%)	2 (3.1%)	
**Anxiety/depression**			
Not anxious/depressed	5 (3.6%)	6 (9.2%)	*p* < 0.001
Slightly anxious/depressed	16 (11.4%)	14 (21.5%)	
Moderately anxious/depressed	55 (39.3%)	31 (47.7%)	
Severely anxious/depressed	38 (27.1%)	10 (15.4%)	
Extremely anxious/depressed	21 (15.0%)	3 (4.6%)	

*The sum might not add up to the total because of missing values*;

* *Wilcoxon signed-rank test*.

#### Recovering Quality of Life (ReQoL-10)

##### Mental Health

Three dimensions of mental health that were most affected: ability to cope, ability to do things and feeling happy (*p* < 0.05, < 0.001, and <0.01, respectively) (Table 2).

**Table 2:** ReQoL frequencies and proportions reported for each mental health question.

**Question**	**Baseline *n* = 140 (100%)**	**Follow-up *n* = 65 (100%)**	*p*-value^*^
**Q1. I found it difficult to get started with everyday tasks**			
None of the time *n* (%)	28 (20.0%)	7 (10.8%)	*p* = 0.572
Only occasionally *n* (%)	18 (12.9%)	12 (18.5%)	
Sometimes *n* (%)	43 (30.7%)	25 (38.5%)	
Often *n* (%)	25 (17.9%)	11 (16.9%)	
Most or all of the time *n* (%)	26 (18.6%)	10 (15.4%)	
**Q2. I felt able to trust others**			
None of the time *n* (%)	33 (23.6%)	9 (13.8%)	*p* = 0.711
Only occasionally *n* (%)	40 (28.6%)	15 (23.1%)	
Sometimes *n* (%)	30 (21.4%)	20 (30.8%)	
Often *n* (%)	20 (14.3%)	14 (21.5%)	
Most or all of the time *n* (%)	17 (12.1%)	7 (10.8%)	
**Q3. I felt unable to cope**			
None of the time *n* (%)	13 (9.3%)	7 (10.8%)	*p* < 0.05
Only occasionally *n* (%)	30 (21.4%)	19 (29.2%)	
Sometimes *n* (%)	44 (31.4%)	24 (36.9%)	
Often *n* (%)	23 (16.4%)	8 (12.3%)	
Most or all of the time *n* (%)	30 (21.4%)	7 (10.8%)	
**Q4. I could do the things I wanted to do**			
None of the time *n* (%)	24 (17.1%)	2 (3.1%)	*p* < 0.001
Only occasionally *n* (%)	18 (12.9%)	12 (18.5%)	
Sometimes *n* (%)	44 (31.4%)	18 (27.7%)	
Often *n* (%)	28 (20.0%)	24 (36.9%)	
Most or all of the time *n* (%)	25 (17.9%)	9 (13.8%)	
**Q5. I felt happy**			
None of the time *n* (%)	18 (12.9%)	6 (9.2%)	*p* < 0.01
Only occasionally *n* (%)	39 (27.9%)	8 (12.3%)	
Sometimes *n* (%)	39 (27.9%)	18 (27.7%)	
Often *n* (%)	28 (20.0%)	23 (35.4%)	
Most or all of the time *n* (%)	16 (11.4%)	10 (15.4%)	
**Q6. I thought my life was not worth living**			
None of the time *n* (%)	61 (43.6%)	27 (41.5%)	*p* = 1.000
Only occasionally *n* (%)	41 (29.3%)	13 (20.0%)	
Sometimes *n* (%)	19 (13.6%)	14 (21.5%)	
Often *n* (%)	11 (7.9%)	7 (10.8%)	
Most or all of the time *n* (%)	7 (5.0%)	4 (6.2%)	
**Q7. I enjoyed what I did**			
None of the time *n* (%)	10 (7.1%)	4 (6.2%)	*p* = 1.000
Only occasionally *n* (%)	24 (17.1%)	8 (12.3%)	
Sometimes *n* (%)	32 (22.9%)	26 (40.0%)	
Often *n* (%)	36 (25.7%)	20 (30.8%)	
Most or all of the time *n* (%)	38 (27.1%)	7 (10.8%)	
**Q8. I felt hopeful about my future**			
None of the time *n* (%)	20 (14.3%)	4 (6.2%)	*p* = 0.093
Only occasionally *n* (%)	28 (20.0%)	11 (16.9%)	
Sometimes *n* (%)	33 (23.6%)	27 (41.5%)	
Often *n* (%)	33 (23.6%)	13 (20.0%)	
Most or all of the time *n* (%)	26 (18.6%)	10 (15.4%)	
**Q9. I felt lonely**			
None of the time *n* (%)	20 (14.3%)	12 (18.5%)	*p* = 0.473
Only occasionally *n* (%)	20 (14.3%)	12 (18.5%)	
Sometimes *n* (%)	40 (28.6%)	16 (24.6%)	
Often *n* (%)	22 (15.7%)	11 (16.9%)	
Most or all of the time *n* (%)	38 (27.1%)	14 (21.5%)	
**Q10. I felt confident in myself**			
None of the time *n* (%)	35 (25.0%)	11 (16.9%)	*p* = 0.110
Only occasionally *n* (%)	34 (24.3%)	10 (15.4%)	
Sometimes *n* (%)	28 (20.0%)	20 (30.8%)	
Often *n* (%)	23 (16.4%)	18 (27.7%)	
Most or all of the time *n* (%)	20 (14.3%)	6 (9.2%)	

*The sum might not add up to the total because of missing values*.

* *Wilcoxon signed-rank test*.

##### Physical Health

Changes in physical health (problems with pain, mobility, difficulties caring for yourself or feeling physically unwell) over the last week were not statistically significant (Table 3).

**Table 3:** ReQoL frequencies and proportions reported for the physical health question.

**Question**	**Baseline *n* = 140 (100%)**	**Follow-up *n* = 65 (100%)**	*p*-value^*^
Please describe your physical health (problems with pain, mobility, difficulties caring for yourself or feeling physically unwell) over the last week			
No problems *n* (%)	54 (38.6%)	17 (26.2%)	*p* = 0.230
Slight problems *n* (%)	30 (21.4%)	16 (24.6%)	
Moderate problems *n* (%)	35 (25.0%)	20 (30.8%)	
Severe problems *n* (%)	14 (10.0%)	8 (12.3%)	
Very severe problems *n* (%)	7 (5.0%)	4 (6.2%)	

* *Wilcoxon signed-rank test*.

##### Quality of Life Changes

**Figure 4:**
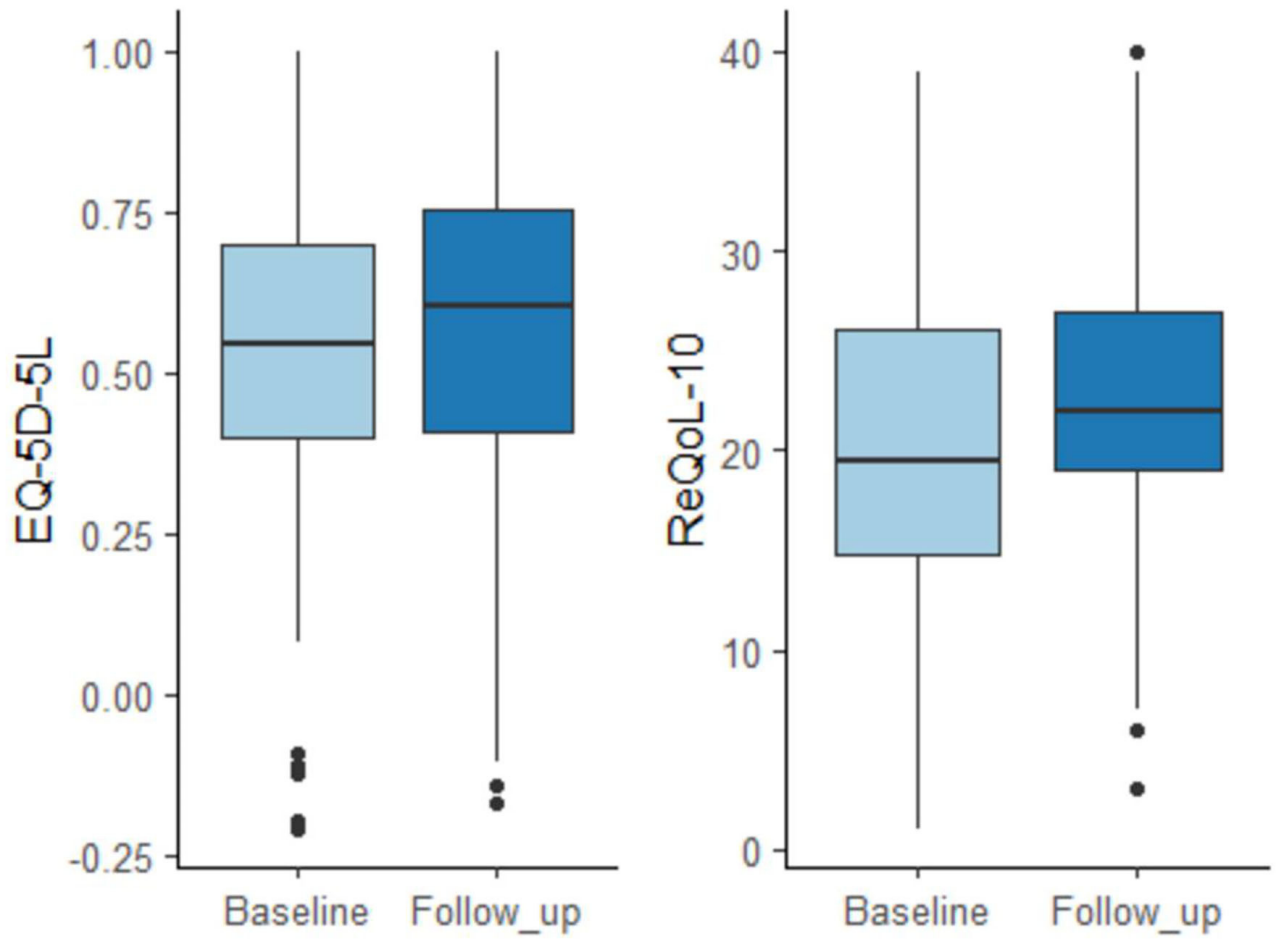
Quality of life changes measured by EQ-5D-5L and ReQoL-10.

Between baseline and follow-up, both of the health related quality of life measures showed slight improvement based on the median scores (Table 4). An improvement by 0.02 points in health status measured by EQ-5D-5L was not statistically significant (*p* = 0.337). ReQoL-10 demonstrated a statistically significant improvement by 1.59 points (*p* < 0.05), however this change was not reliable according to the recommended interpretation of ReQoL-10 scores. The minimum important difference of 5 points is the smallest change in a ReQoL-10 score that is considered clinically or practically important. Figure 4 shows this information graphically.

**Table 4:** Quality of life changes measured by EQ-5D-5L and ReQoL-10.

	**Overall index score [95% Conf. Interval]**		
	**Baseline**	**Follow-up**	Diff, *p*-value^*^
EQ-5D-5L	0.53 [0.49; 0.57]	0.55 [0.49; 0.63]	0.02, *p* = 0.337
ReQoL-10	20.39 [19.02; 21.70]	21.98 [20.05; 23.92]	1.59, *p* < 0.05

* *Wilcoxon signed-rank test*.

##### Employment Measures (Occupational Status and Absenteeism)

**Figure 5:**
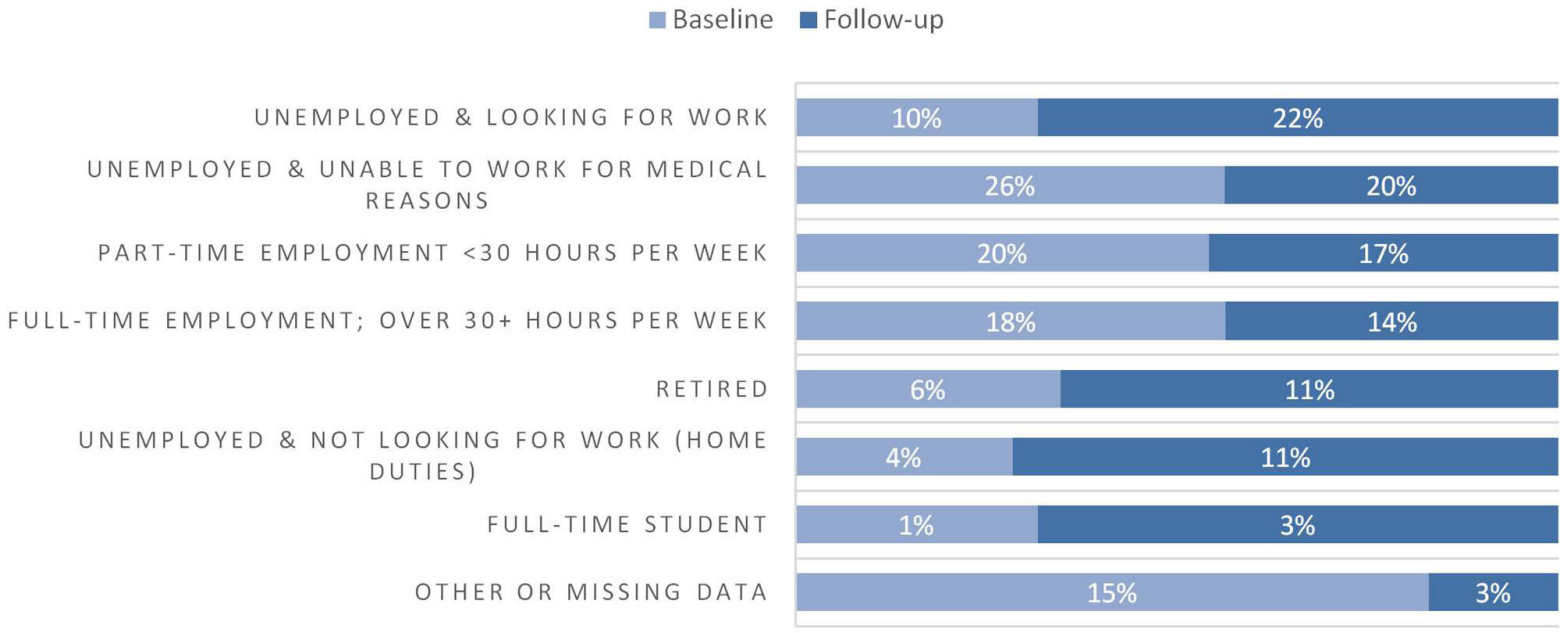
Occupational status at the baseline and follow-up.

Changes in the self-reported employment measures were inconclusive. While the number of people looking for work has increased in relative terms from 10% (*n* = 14) to 22% (*n* = 14) and the number of people unable to work for medical reasons has dropped from 26% (*n* = 36) to 20% (*n* = 13); the number of employed people has dropped from 18% (*n* = 25) to 14% (*n* = 9) for full-time employment and from 20% (*n* = 28) to 17% (*n* = 11) for part-time employment (Figure 5).

Those who identified as being employed, reported a greater absenteeism from work over the past 4 weeks (28 days) at the follow-up assessment compared to the baseline. However, these results should be interpreted cautiously due to the small sample (Table 5).

**Table 5:** Absenteeism (in hours) in the past 4 weeks (28 days).

	**Baseline(*n* = 25)**	**Follow-up(*n* = 5)**	**Diff**
Full-time employment; over 30+ h per week, h	1.4	1.6	0.2
Part-time employment < 30 h per week, h	2.6	3.0	0.4

## Discussion

The process evaluation used the CTW experience to better understand the process of implementing a new service like CTW and to identify potential outcome measures that could be incorporated into routine data collection tools in the future. The hybrid research design provided a conceptual framework for evaluating the implementation process while testing outcome measures that could monitor and assess client-reported outcomes of the CTW service (21). Further, for CTW, the QI approach supported the hybrid research design enabling findings from the evaluation to be quickly integrated into the CTW service.

### Improving the Accessibility of Mental Health Services

The process evaluation provided evidence that CTW had improved referral processes, that eligible people are no longer declined access, and that waitlists have been removed which is a significant achievement given the long waitlists prior to CTW commencing. Therefore, the process evaluation suggested that CTW were connecting the right people, to the right service, at the right time, as far as was practicable.

In addition, enablers for the CTW service, such as the ability to adapt to internal and external impacts, workforce flexibility, and building relationships with other service providers, created opportunities for CTW to broadened the scope and type of psychological services offered to eligible people living in North Queensland (e.g., Group Therapy). Where barriers were typical to the environment in which CTW operated (e.g., lack of service providers for young people in some areas) innovative solutions helped CTW to adapt to their environment, and helped to continue with the work of connecting clients with suitable services (e.g., Telehealth). Comprehensive service mapping meant the NQPHN was better informed about areas where there were inadequate numbers or types of mental health service providers. While the small sample size limited our understanding from the client perspective, this process evaluation contributes to our understanding about the implementation process. Given the scarcity of published research about the process of implementing and operating stepped care mental health referral services, these findings contribute to the empirical evidence-base (4, 6).

The process evaluation was useful for understanding the referral pathways, and the enablers and barriers for implementation. However, the process data could not show the extent to which the CTW service was impacting on the client's health and well-being, let alone whether the service was value for money. Hence, the assessment to identify potential outcome measures, which was conducted at the same times as the process evaluation, was a valuable addition to the overall study. Particularly, in terms of identifying suitable measures for determining client outcomes and value for money for future evaluations.

### Potential Outcome Measures

This section discusses the findings to identify suitable outcomes measures for future research. The three outcome measures piloted in this study were the EQ-5D-5L, ReQoL-10 and specific questions about employment and absenteeism. The analysis found that both the EQ-5D-5L and ReQoL-10 showed “mixed” changes in health: better on one dimension, but worse on another. Neither of the measures were found to be superior.

Both measures showed slight improvements based on the median scores. However, improvement in health status by 0.02 points measured by EQ-5D-5L was not statistically significant (*p* = 0.337). ReQoL-10 demonstrated a statistically significant improvement by 1.59 points (*p* < 0.05), but this change was not clinically meaningful according to the recommended interpretation of ReQoL-10 scores requiring a minimum of 5 points. Changes associated with employment (self-reported occupational status and absenteeism) had the largest number of missing data and were inconclusive due to small sample size. It is recommended that the service continues collecting these data until longer term data on clients' outcomes and cost-effectiveness becomes available.

Further, the study found that eleven CTW clients (17%) were triaged to alternative services, including some outside the CTW network. Therefore, it is likely that the health outcomes for these clients were not measured in this evaluation. However, triaging referrals to other sources that better met their needs is an intended feature of the CTW service design and as far as the process is concerned is consistent with the objective to connect the right person, to the right service, at the right time. Hence, it is through the hybrid design, and more specifically the analysis of the potential outcomes measures that a more nuanced understanding of how to measure the effectiveness of the CTW service began to emerge.

### Insights

The insights gained from the process evaluation of a real-world organization, such as the one conducted with CTW, not only contributes to the scarcity of empirical evidence in this area, it is consistent with the principles of implementation science research (27, 28). In the past 20 years, implementation science has supported the use of theoretical frameworks to help researchers and health professionals to better understand why a given intervention succeeded or failed (28, 29). Therefore, using the CTW experience as a standalone case in a specific context contributes to the empirical literature as well as providing valuable insights for health managers and organizations, particularly those considering how to implement a referral hub for stepped care mental health services. Despite the limitations, this study provides valuable insights from an empirical study, using a hybrid study design.

The evaluation highlighted the barriers and enablers in the process of service implementation. Some of the barriers to service implementation reduced access to the type, and quantity of data that could be collected for an outcome and economic evaluation. These findings are consistent with the literature where a general lack of appropriate and compelling data for outcome evaluations is typical. While we identified some implementation science studies (30) and evaluations of stepped care in the areas of treating smoking dependence, maternal mental health and anxiety disorders, we were unable to locate outcomes evaluations for mental health services referral hubs similar to CTW in the literature. This highlights the need for evaluations, such as this one which seek to identify how the type of data needed for outcomes evaluations can be collected in the course of routine service provision, and the benefits of a QI approach for the process of implementing a new service. Therefore, the hybrid research design provided a sound methodology for the objectives of this study.

While, the barriers to the process of implementing CTW and in collecting suitable outcomes data created some limitations for the study; it also provided insights about conducting process and outcomes evaluations with new real-world services, particularly in an area, such as mental health, where positive health impacts can take time to be achieved (7, 8). Further, there is complexity in measuring the outcomes of a referral service model that involves two distinct activities: (1) assessment and referral to an appropriate service; and (2) provision of psychological therapy (or other intervention). While CTW oversaw the assessment and referrals, the therapy was delivered by around 100 individual service providers or organizations. This study was limited to CTW and therefore had no insight into the type or quality of the services provided by the individual service provider (e.g., psychologist), that likely had a major influence on the overall outcomes for individual clients. Hence, the identification of potential outcomes measures is a first step in understanding how to measure the effectiveness of a referral service such CTW.

#### Lessons Learned

The insights from this study included a summary of the lessons learned that can inform similar studies in regard to co-creation research, evaluating referral services, and insight into generating suitable data for outcomes analysis. Firstly, the QI PAR approach was an inclusive and reflective environment for the researchers, CTW staff, carer and client representatives, and management. In this type of environment, it was possible to co-create narrative about the process of implementing a mental health referral service that to the authors' knowledge does not currently exist in the literature. Secondly, the review of the existing data and potential data sources highlighted the need to determine measures to evaluate the benefit of CTW which is separate, yet related, to the consumer outcomes arising from therapeutic care. The evaluation found that a general consumer experience survey does not generate the type of data needed to measure the role of the referrer in the client experience. Finally, if referral hubs that assess and refer clients to mental health services are to be evaluated for effectiveness, cost-efficiencies and value for money, there is a need to generate consistent longitudinal data so that it is possible to conduct comparisons. Therefore, there needs to be agreement on the indicators of service provision available in routine datasets.

### Limitations

Many of the limitations for this study were associated with the challenges of evaluating the process of implementing a new service in real time, and in auditing the client reported outcomes during a period when processes and systems were being developed. The aforementioned challenges led to delays with data collection, and a high dropout rate for the follow-up assessments which resulted in missing data for the assessment of potential outcomes measures. As a result there was a low response rate for the YESS survey, and a small sample size which was a limitation for the assessment of potential outcome measures. The lack of baseline data on age and gender, and severity of disease was also a limitation for this study.

It is acknowledged that changes in the client outcomes may or may not be due to the CTW service received. However, as far as the assessment of potential outcomes measures is concerned, this study is a proof of concept designed to pilot a set of selected client-reported outcome measures for the purposes of a QI process.

## Conclusion

The evaluation suggested that implementation of CTW, a centralized assessment and referral service, improved timely access to psychological therapies for people with moderate to severe mental health needs in North Queensland (Australia). Given the scarcity of empirical evidence about the process of implementing new mental health referral services, this research makes a valuable contribution by evaluating the implementation process and in identifying the barriers and enablers for the implementing a new mental health referral service. Further, this evaluation provides evidence about the real-world benefits of the hybrid research design. The hybrid design, together with the QI approach for collecting data about CTW's processes and client outcomes provided a rapid feedback loop that informed the planning and implementation processes for CTW. In addition, it provided insight into how the collection of data suitable for an outcomes evaluation can be incorporated into routine data collection practices to build larger datasets on which to make more informed decisions about the efficiency and effectiveness of services, such as CTW.

## Data Availability Statement

The data analyzed in this study is subject to the following licenses/restrictions: the non-identifiable datasets used for this study are not publicly available. Requests to access these datasets should be directed to Komla Tsey, komla.tsey@jcu.edu.au.

## Ethics Statement

The studies involving human participants were reviewed and approved by James Cook University (JCU) Human Research Ethics Committee (HREC) (#H7549). Written informed consent for participation was not required for this study in accordance with the national legislation and the institutional requirements.

## Author Contributions

LO completed the literature search. PE and JPe co-ordinated the data collection. LO and IK analyzed the data. LO, IK, KT, and JPr contributed to the first draft. PE and JPe provided the industry experience and contributed to subsequent drafts. All authors contributed to the conception and design of this article, contributed to the interpretation of the analyzed data and reported findings, contributed, read, and approved the submitted manuscript.

## Conflict of Interest

LO, IK, JPr, and KT declare that they received funding to conduct the research from Neami National. PE and JPe declare that they are employed by Neami National and JPe also declares that they led the implementation of the Connect to Well-being service in North Queensland. We all declare that the financial relationships supported the research; however, they have not influenced the submitted work.
