# Supplementary material for: Evaluating the Implementation of a Mental Health Referral Service “Connect to Wellbeing”: A Quality Improvement Approach
Source: Front Public Health. 2020 Dec 14;8:585933. doi: 10.3389/fpubh.2020.585933 (PMC7767852; doi:10.3389/fpubh.2020.585933)
Supplement: Supplementary file 1 [file Data_Sheet_1.PDF]

Supplementary Figure S1: Evaluation Plan

Research Question	Objectives	How will we know?	Data source	Measures/Indicators	Outcomes
1. What are the barriers to, and factors enabling, the process of implementing the CTW service?	▪ Understand the extent to which CTW connects people in need of services to the right service at the right time<br>▪ Process of implementing the new service	▪ Clarify and articulate the assumptions / evidence underpinning the theory of change including process and outcome indicators for assessing service performance	▪ A review of CTW program logic in the context of relevant Stepped Care Model of mental health<br>▪ Documents from 19 July evaluation planning workshop	▪ Access and analyse additional data from new outcome measures<br>▪ Conduct an economic evaluation	▪ Best practice Stepped Care Model, tailored to needs of clients<br>▪ A longer-term sustainable evaluation framework including process, impact and cost indicators
	▪ Identify the barriers and enablers to effective CTW service implementation and actions/strategies to improve the service quality and outcomes	▪ Routinely analyse and present service performance data (process and outcome) to CTW staff / governance group for critical reflection and action towards QI	▪ Intake, assessment, and referral data<br>▪ Records of QI meetings / session with CTW staff / governance group and stakeholder meetings		▪ A CTW psychological service that is underpinned by a culture of data-driven QI
2. What are the outcome measures that the CTW service can collect through routine data collection to inform quality improvement?	▪ Extent to which clients /families are satisfied with the CTW service<br>▪ Impact of the CTW service on the psychological wellbeing of clients<br>▪ Effectiveness of CTW on health and wellbeing outcomes compared with the previous service.	▪ Routinely analyse and present service performance data (process and outcomes) to CTW staff / governance group for critical reflection and action towards quality improvement (QI)	▪ Intake, assessment and referral data	▪ Access and analyse additional data from new outcome measures	▪ Increased satisfaction among clients/families<br>▪ Increased psychological wellbeing among clients<br>▪ Impact of CTW on service access; impact of CTW on client/family wellbeing; an understanding of the enablers of successful implementation
3. How can insights gained from the CTW experience inform future service development?	▪ The enablers and potential pitfalls of implementing the CTW model	▪ Reflections / feedback from CTW staff / governance groups during QI sessions and stakeholder meetings<br>▪ Extent to which the project findings have been used to inform practice within and beyond the CTW service	▪ Meetings, reports and publications<br>▪ Evidence of research uptake such as the use of the project results to support new grant applications	▪ Access and analyse additional data from new outcome measures<br>▪ Conduct an economic evaluation	▪ Improved understanding of the enablers and benefits of the CTW approach to improving service access and resource use<br>▪ Evaluation of CTW which can inform future service development
