# Supplementary material for: Evaluating the Implementation of a Mental Health Referral Service “Connect to Wellbeing”: A Quality Improvement Approach
Source: Front Public Health. 2020 Dec 14;8:585933. doi: 10.3389/fpubh.2020.585933 (PMC7767852; doi:10.3389/fpubh.2020.585933)
Supplement: Supplementary file 2 [file Data_Sheet_2.PDF]

Supplementary Table S1: Steps in the evaluation process

Steps	Key Activities
Planning the evaluation	▪ One-Day workshop with the evaluation collaboration to co-design the Evaluation Plan<br>▪ Obtained the relevant ethics approvals<br>▪ Set up an Evaluation Steering Group including consumer, CTW staff and carer representation
Literature reviews	▪ Conducted a scoping literature review to refine the evaluation design<br>▪ Conducted a rapid literature review to identify current knowledge about specific aspects of the evaluation
Development of Evaluation plan	▪ Identified potential outcomes measures through a scoping analysis of the existing data to determine appropriateness as process and outcome measures
Inaugural Evaluation steering committee meeting	▪ Establish ground rules for the committee meetings<br>▪ Review outcomes of the scoping literature and data review<br>▪ Based on current evidence and available data - decision made to revise the Evaluation Plan to include economic evaluation and to extend the evaluation to include at least three QI cycles.
Revised Evaluation Plan	▪ Implementation of the three evaluation cycles:<br>- Baseline: July 2018 to November 2018<br>- Cycle One: December to April 2019<br>- Cycle Two: May to August 2019<br>- Cycle Three: August to December 2019
Second Evaluation steering committee meeting	▪ Revised Evaluation Plan tabled, including the proposed new survey tools.<br>▪ Analysis of the emergent data presented and refinements to the data collection tools and methods discussed.
Third Evaluation steering committee meeting	▪ Reviewed and discussed the insights from CTW's year one report<br>▪ Facilitated reflective discussion about CTW implementation process, and the evaluation process
Data collection, analysis and interpretation	▪ Thematic analysis of the qualitative data and analysis of the quantitative data conducted for the Process Evaluation.<br>▪ Analysis of the quantitative data conducted for the Assessment of potential outcome measures<br>▪ Interpretation of findings and Final Report drafted
Final Evaluation steering committee meeting	▪ Draft Final Report tabled, findings discussed. Final reflections on the process and outcomes evaluation, and plans made for QI activities and future research/evaluation priorities.
